# Effect of Rare Earth on Microstructure and Wear Resistance of In-Situ-Synthesized Mo_2_FeB_2_ Ceramics-Reinforced Fe-Based Cladding

**DOI:** 10.3390/ma13163633

**Published:** 2020-08-17

**Authors:** Jun Jin, Junsheng Sun, Weimin Wang, Jijun Song, Hu Xu

**Affiliations:** Key Laboratory for Liquid-Solid Structural Evolution and Processing of Materials, Ministry of Education, Shandong University, Jinan 250061, China; 201733689@mail.sdu.edu.cn (J.J.); weiminw@sdu.edu.cn (W.W.); 200799013860@email.sdu.edu.cn (J.S.); xh0714@mail.sdu.edu.cn (H.X.)

**Keywords:** surfacing, Mo_2_FeB_2_, rare earth Y, refining effect, wear resistance

## Abstract

Mo_2_FeB_2_ ceramics-reinforced Fe-based cladding with various rare earth (RE) concentrations were prepared by the carbon arc surfacing process. The effects of RE content on the microstructure, phase composition, hardness and wear resistance of the cladding were systematically discussed. Meanwhile, the area fraction and grain size of Mo_2_FeB_2_ phase were exactly measured. Moreover, the refining mechanism of rare earth Y was analyzed. Results revealed that the claddings consisted of Mo_2_FeB_2_, FeCr, MoB and CrB. Adding the rare-earth Y decreased the grain sizes of Mo_2_FeB_2_ phase. Furthermore, grain-refining effects of Mo_2_FeB_2_ phase were significant when the RE content was 2% and hard phases evenly distributed in the cladding. In addition, the maximum microhardness value of claddings was about 1078 HV. The claddings with 2% RE contents had better wear resistance, which was equivalent to a sintered sample.

## 1. Introduction

The ternary boride cermets, such as Mo_2_FeB_2_, Mo_2_NiB_2_ and WCoB, showing higher corrosion resistance and thermal stability at high temperatures, are recognized as promising materials for wear-resistant applications [1,2,3,4]. In particular, Mo_2_FeB_2_-based cermets have been widely applied to screw, barrel and other construction machinery owing to their low cost, superior wear resistance, corrosion resistance, chemical stability, approximate thermal expansion coefficient and perfect metallurgical bonding with steels [5,6,7,8,9].

In general, Mo_2_FeB_2_-based cermets are synthesized by reactive boronization sintering, Yu et al. [10,11] have obtained Mo_2_FeB_2_-based cermets using the sintering process and pointed out that sintering temperature can influence the morphology of the Mo_2_FeB_2_ phase. In addition, the effect of alloying elements on their densification behavior, microstructure and properties have been systematically investigated [12,13,14,15]. However, it is worth mentioning that expensive equipment and complex processes would increase costs and limit applications of Mo_2_FeB_2_-based cermet. At the same time, the preparation of Mo_2_FeB_2_-based claddings using welding techniques has rarely been studied. Welding has its own advantages such as simple operation and low costs contrasted with the sintering process. However, Mo_2_FeB_2_ growing from weld pool makes Mo_2_FeB_2_ have a large size and many problems such as insufficient hardness, large brittleness and poor wear resistance.

Rare earth (RE) elements have the ability to reduce the effect of impurity by forming intermetallic compounds and purify the melt [16]. In addition, RE elements can reduce the amount of second phases and grain size, resulting in a more homogeneous and refined microstructure [17]. Wang et al. [18] studied the effect of La_2_O_3_ on friction and wear properties of laser cladding Ni-based coatings and obtained that the microstructure of Ni60 cladding layers with added rare-earth oxides are obviously refined. Feng et al. [19] investigated the effect of LaB_6_ addition on the microstructure and properties of (Ti_3_Al+TiB)/Ti composites and pointed out that the microstructure was refined and the second phase reinforcements were distributed more homogeneously with appropriate LaB_6_ addition (3.0%). However, there have been few studies on the mechanism through which RE elements affect the Mo_2_FeB_2_-based claddings. Therefore, it is necessary to focus on the effects of RE elements on the Mo_2_FeB_2_-based claddings. In this study, the rare-earth alloy was added to Mo_2_FeB_2_-based claddings, which is used to compare the grain-refining effects, investigate rare-earth Y in the Mo_2_FeB_2_-based claddings, determine the influence and mechanism of the rare-earth Y and analyze the influence of rare-earth Y on the hardness and wear resistance of cladding. The research in this study is of great significance for the preparation and application of high-performance rare-earth Mo_2_FeB_2_-based claddings.

## 2. Materials and Methods

Mo powders, FeB powders, RE-Mg-Si powders (RE), Cr powders and carbonyl Fe powders were used as raw materials (Jinzhou Hongda New Material Co., Ltd., Jinzhou, China). Table 1 shows the chemical composition and particle size of the alloy powders. These powders were mixed in QM-3SP2 planetary ball mill (Shanghai Shupei Experimental Equipment Co., Ltd., Shanghai, China) for 6 h at a speed of 580 r/min. After milling, the powders were pelletised with 10% sodium silicate, and pressed into the thin alloy blocks (90 mm × 30 mm × 3 mm) in FK2000 briquetting machine (Weihai Sandun Welding Material Engineering Co., Ltd., Weihai, China) at a pressure of 60 MPa. The density of the blocks was 5.08 g/cm^3^ and Table 2 shows the composition of alloy blocks. The blocks remained at room temperature for 6 h and then dried in an oven (YCH-30KG, Wujiang Rongshun Electric Heating Equipment Co., Ltd., Suzhou, China) at 200 °C for 1 h. Q235 steel (200 mm × 50 mm × 10 mm) was selected as base metal and its chemical composition (wt.%) is 0.15% C, 0.5% Mn, 0.3% Si—both S and P do not exceed 0.045%. Mo_2_FeB_2_-sintered samples were obtained from Dongguan Jieyu Machinery Co., Ltd. (Dongguan, China).

**Figure 1:**
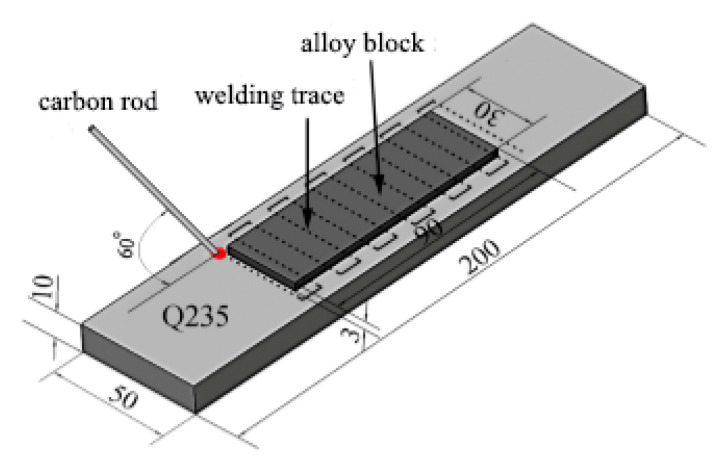
Schematic diagram of the carbon arc surfacing (Unit: mm).

Alloy blocks was placed on the surface of Q235 steel substrate using ZX7-400 STG carbon arc welding machine (Shandong Aotai Electric Co., Ltd., Jinan, China). A graphite rod was held with a welding torch so that an arc was formed between the graphite rod and the substrate. The alloy block melted into the weld pool formed on the substrate, thereby reducing the burning loss of the alloy elements. The technological parameters of the surfacing process given in Table 3. After that, the second layer was fabricated with the same optimized parameters. Figure 1 illustrates schematic diagram of the carbon arc surfacing.

After welding, the cladding was cut into a typical cross section. Then, the samples were ground with abrasive papers and polished by 1.5 μm diamond paste before observation. Subsequently, the specimens were etched using a mixed acid solution (HF: 20 vol.%, HCL: 30 vol.%, HNO_3_: 50 vol.%) within 12~14 s. The microstructure was studied with scanning electron microscope (SEM) (JSM-6600V, Japanese electronics company, Tokyo, Japan) in backscattered electron (BSE) mode. While analysis of the chemical composition of both the hard-phase and eutectic matrix was performed by energy dispersive spectroscopy (EDS) linked to SEM, X-ray diffraction (XRD) line profiles were measured using an x-ray diffractometer with Cu Kα radiation (λ = 0.154056 nm) and the scanning rate was set as 8°/min with a scan step of 0.02°. Phase fraction and grain size were measured by Image-Pro Plus 6.0 (IPP 6.0) software (National Institutes of Health, Bethesda, America). In addition, electron probe micro-analysis (EPMA-JXA-8530F PLUS, Japanese electronics company, Tokyo, Japan) was employed to investigate the existence and distribution of rare-earth Ce, Y elements in the Mo_2_FeB_2_ claddings.

Microhardness along the depth in cross-sections of claddings was measured using micro-Vickers hardness tester (DHV-1000, Shanghai Shangcai Testing Machine Co., Ltd., Shanghai, China) with a load of 500 g for 10 s dwell time. The wear resistance test was conducted on M-2000 testing machine (Hebei Xuanhua Zhengli Balancing Machine Co., Ltd., Hebei, China) by a block on ring wear tester under dry and rotating conditions in room temperature. The samples were machined to 31 mm × 7 mm × 5 mm by wire-cut electrical discharge machining (EDM) (DK7732, Taizhou Tianlong CNC Machine Tool Co., Ltd., Taizhou, China). Then, the samples were ground with abrasive papers to remove metallic oxide off the surface, which smoothed the sample surface. Carburized 20CrMnTi steel with the size of 40 mm in diameter and 10 mm in thickness was employed as the counterpart, which had a Rockwell hardness of 60.2 HRC. The load, the rotating speed and wear time were 1.5 × 10^2^ N, 4.0 × 10^2^ r/min and 60 min, respectively. As comparison, Mo_2_FeB_2_-sintered samples were also given a wear-resistance test under the same conditions. The wear weight-loss value of the specimens was calculated by averaging three measurements. Before the weight-loss measurements, samples were washed with ultrasonic cleaning apparatus and dried. Alongside, the wear morphology of the claddings and sintered samples were observed by SEM.

## 3. Results and Discussion

### 3.1. Microstructure and Composition

**Figure 2:**
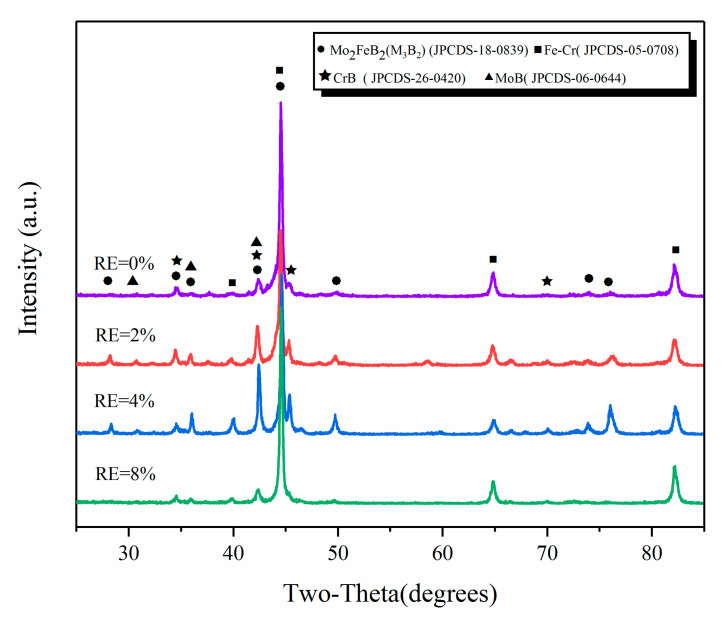
XRD analysis of claddings with different RE content.

Figure 2 gives the XRD patterns of different RE-contents claddings. As can be seen from Figure 2, the claddings consist of Mo_2_FeB_2_, M_3_B_2_ (M: Mo, Fe, Cr et al.), FeCr and binary borides such as MoB and CrB. It should be noted that M_3_B_2_ is a complex ternary boride (Fe,Mo,Cr)_3_B_2_ formed by Mo_2_FeB_2_ and Cr at high temperature [20,21,22].

**Figure 3:**
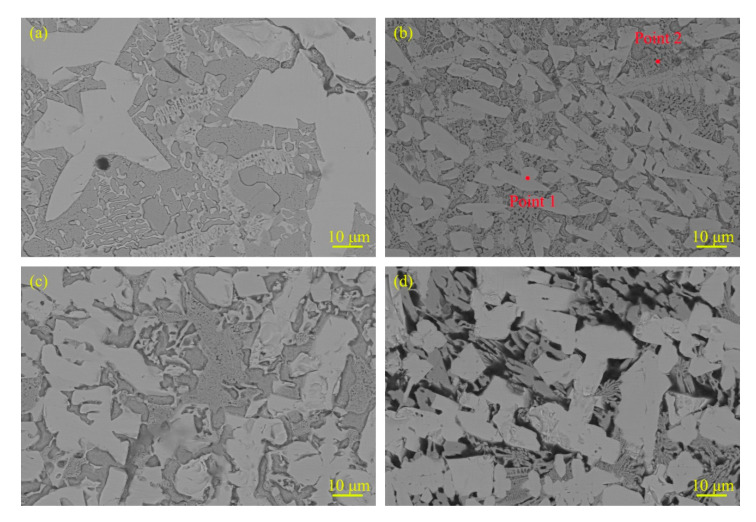
Microstructural morphology of claddings with different RE contents. (**a**) RE = 0%; (**b**) RE = 2%; (**c**) RE = 4% and (**d**) RE = 8%.

Figure 3 shows the typical microstructure with different RE contents. The cladding is mainly composed of white hard phases and eutectic structure and the RE content has distinct influence on the microstructure of claddings. The number of white hard phases of cladding with RE contents remarkably increases compared with the cladding without RE content. Moreover, the hard phases in cladding are significantly refined when the RE content is 2%. As the RE contents increase, the hard phases begin to grow coarse. When the RE content arrives at 8%, the hard phases are so coarse that some of them connect to each other. This is because the nucleation rate of the system will increase as the content of rare-earth elements increases [23]. At the same time, according to the dissolution and diffusion mechanism [24], small hard-phase particles dissolve during the arc thermal cycle and Mo and B precipitate on the originally coarse Mo_2_FeB_2_ hard particles, which serve as nucleation centers and gradually grow to connect to each other. Moreover, the composition of hard phase and eutectic structure of the cladding with 2% RE content were analyzed by EDS. The results of EDS are listed in Table 4. It should be noted that the B content were not accurate because of the insensitivity of EDS to B element. Point 1 and point 2 in Figure 3b are used for spot scans of the hard phases and eutectic structure, respectively. From Table 4, it was found that there was Mo, Fe, Cr and B in the hard phase and eutectic structure. However, it obviously showed that Mo content was enriched in white hard phases, and Fe content was concentrated in the eutectic structure. Combining with XRD and EDS results, the white hard phases are Mo_2_FeB_2_ and M_3_B_2_ complex boride type.

**Figure 4:**
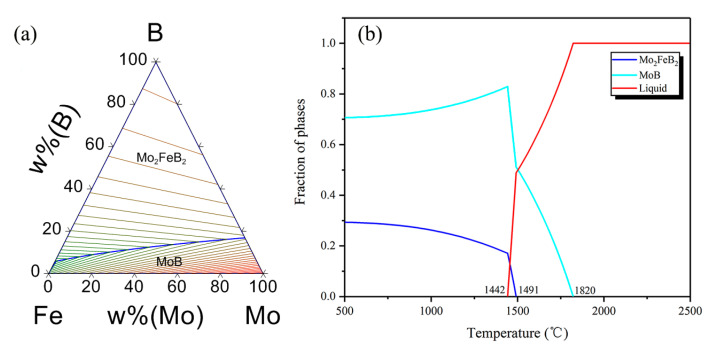
Calculated liquidus surface projection of Mo-Fe-B system and Molar fraction variation of each phase. (**a**) Liquidus surface projection; (**b**) molar fraction variation of each phase.

To further analyze the formation mechanism of Mo_2_FeB_2_ hard phase in claddings, CALPHAD modelling of multicomponent Mo-Fe-B systems was employed using Pandat software (Pandat 2020, CnTech Company, Beijing, China) [25]. The Cr compounds were not considered. The SGTE thermodynamic database was used for alloy systems [26]. Figure 4 shows the calculated liquidus surface projection of Mo-Fe-B system and Molar fraction variation of each phase. From Figure 4b, the MoB phase precipitates from the liquid phase at 1820 °C (L→MoB). At 1491 °C, the molar fraction of the liquid phase decreases sharply and the MoB phase of that continues growing. The molar fraction of the Mo_2_FeB_2_ phase starts to increase, which illustrates that the Mo_2_FeB_2_ phase begins to precipitate. It should be noted that Mo_2_FeB_2_ phase has two types of forming methods. Firstly, the Mo_2_FeB_2_ phase precipitates from the liquid phase (L→Mo_2_FeB_2_) [27]. Secondly, MoB reacts with Fe to form the Mo_2_FeB_2_ phase (2MoB+Fe→Mo_2_FeB_2_) [28]. Therefore, the Mo_2_FeB_2_ phase could form according to these methods at 1491 °C. As temperature decreases, the liquid phase diminishes until it disappears at 1442 °C. In this temperature range, the liquid phase, the MoB phase and the Mo_2_FeB_2_ phase can coexist. In addition, Wang et al. [27] calculated the variation of Gibbs free energy of Mo-Fe-B-Cr system in the weld pool at 1389 °C and pointed out that the Gibbs free energy of MoB and CrB were −136 KJ/mol and −59.581 KJ/mol, respectively, illustrating that MoB and CrB phases can exist in high temperature, liquid phase, which was consistent with the XRD results (Figure 2).

### 3.2. Statistics of Phase Fraction and Grain Size

**Figure 5:**
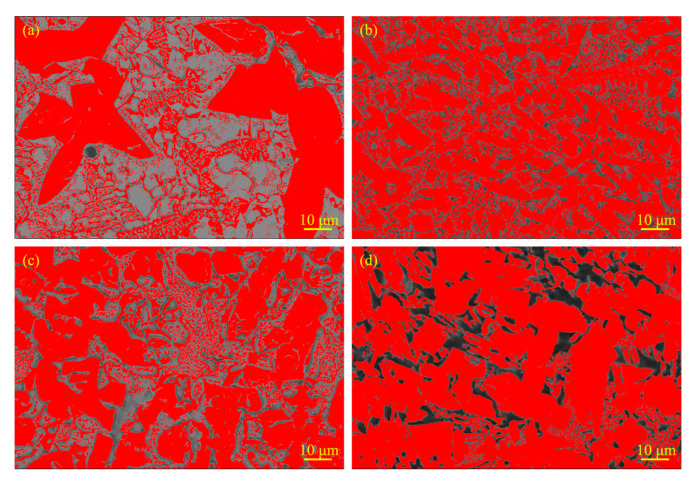
Images of calculation of hard phase fraction: (**a**) RE = 0%; (**b**) RE = 2%; (**c**) RE = 4% and (**d**) RE = 8%.

As for the statistics of phase fraction and grain size, 20 images of each sample were used for the determination and here we just chose the most representative images to show. Figure 5 shows the images of hard-phase fraction. The light color indicates the Mo_2_FeB_2_ phase while the dark color belongs to the matrix phase. When the RE content is 0%, 2%, 4% and 8%, the area fraction of Mo_2_FeB_2_ hard phase is 59%, 72%, 67% and 74%, respectively. The addition of rare-earth elements increases the amount of Mo_2_FeB_2_ hard phase, so that the area fraction of Mo_2_FeB_2_ hard phase in the cladding with RE content is as high as 67% or more. Moreover, when the RE content is 2% (Figure 5b), the Mo_2_FeB_2_ phase is refined and area fraction reaches 72%, which is 14% higher than the cladding with 0% RE content.

**Figure 6:**
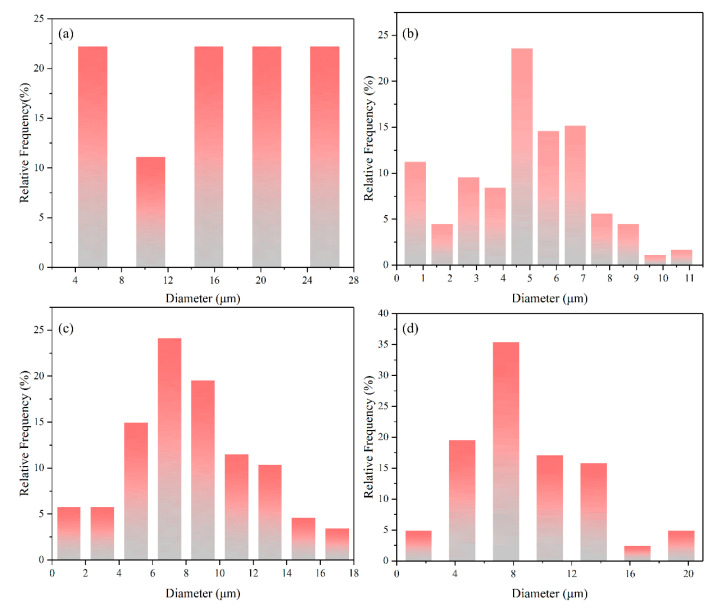
Grain size distribution of Mo_2_FeB_2_ hard phases with different RE contents: (**a**) RE = 0%; (**b**) RE = 2%; (**c**) RE = 4% and (**d**) RE = 8%.

Figure 6 shows grain size distribution of Mo_2_FeB_2_ hard phases with different RE contents. When the RE content is 0%, average grain size of the Mo_2_FeB_2_ hard phase is 16.52 μm and mainly distributed in 14~25 μm, as shown in Figure 6a. In terms of 2% RE content, average grain size of the Mo_2_FeB_2_ hard phase is 4.78 μm and primarily distributed in 4.2~5.2 μm (Figure 6b). When the RE content is 4%, average grain size of the Mo_2_FeB_2_ hard phase is 8.2 μm and principally distributed in 6~8 μm, as shown in Figure 6c. As for 8% RE content, average grain size of the Mo_2_FeB_2_ hard phase is 8.88 μm and chiefly distributed in 6~9 μm (Figure 6d).

**Figure 7:**
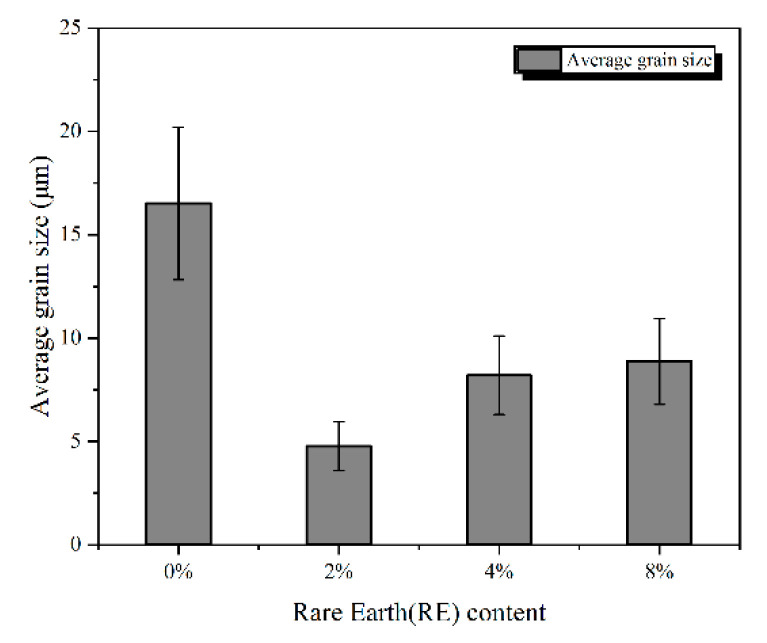
Average grain size of Mo_2_FeB_2_ hard phases with different RE contents.

Figure 7 shows the effect of RE content on grain size and size variance of the Mo_2_FeB_2_ hard phases. From Figure 7, the average grain size of the Mo_2_FeB_2_ hard phases is significantly refined and the size variance is smaller with adding RE. When the RE content is 2%, the size of the Mo_2_FeB_2_ hard phases is 1/3.44 of the cladding with 0% RE content, the smallest variance, which is the best addition for the refinement.

### 3.3. Refining Mechanism of Rare Earth

**Figure 8:**
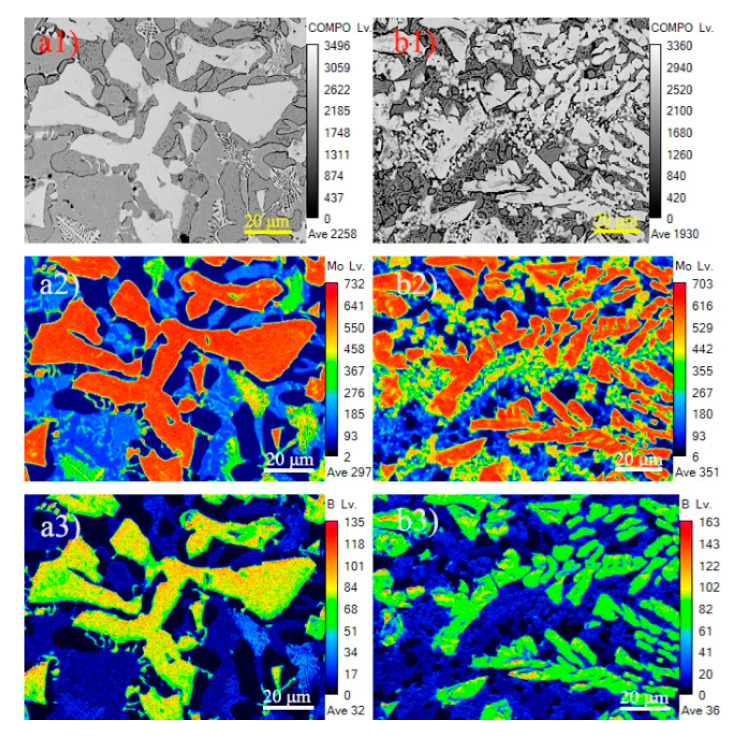

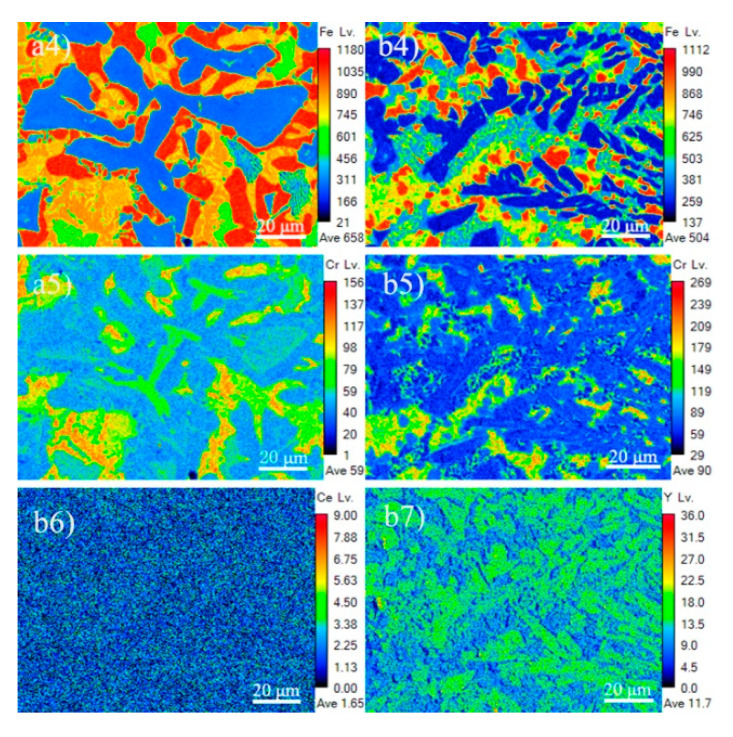
Elements distribution image of cladding with different RE contents: (**a1**) BSE images of cladding with 0% RE content; (**a2**) Mo element distribution image of cladding with 0% RE content; (**a3**) B element distribution image of cladding with 0% RE content; (**a4**) Fe element distribution image of cladding with 0% RE content;(**a5**) Cr element distribution image of cladding with 0% RE content; (**b1**) BSE images of cladding with 2% RE content; (**b2**) Mo element distribution image of cladding with 2% RE content; (**b3**) B element distribution image of cladding with 2% RE content; (**b4**) Fe element distribution image of cladding with 2% RE content; (**b5**) Cr element distribution image of cladding with 2% RE content; (**b6**) Ce element distribution image of cladding with 2% RE content; (**b7**) Y element distribution image of cladding with 2% RE content.

To further study the mechanism of RE content on Mo_2_FeB_2_ hard phases, the electronic probe (EPMA) was employed to analyze element distribution of samples. Figure 8 presents the elements’ distribution of cladding with 0% and 2% RE contents. Mo and B elements are mainly enriched in Mo_2_FeB_2_ hard phases, as shown in Figure 8a2—Mo, Figure 8a3—B and Figure 8b2—Mo, Figure 8b3—B. While Fe element is primarily distributed in the matrix and eutectic structure (Figure 8a4—Fe and Figure 8b4—Fe), and Cr element is principally concentrated in the matrix, which guarantees that the matrix has certain corrosion resistance [12]. Figure 8b6—Ce and Figure 8b7—Y are the distribution images of the Ce and Y elements. The distribution of Ce element is relatively uniform and there is no obvious segregation phenomenon, while the Y element is enriched in Mo_2_FeB_2_ hard phases. Elemental Y has higher melting point (1522 °C)—Liu [29] found that Y can be used as the nucleation particle of Mg_2_Si and refining the grains of Mg_2_Si. The Y element is segregated in the Mo_2_FeB_2_ hard phases (Figure 8b7—Y), which can be used as potential hard-nucleation particle of the Mo_2_FeB_2_ phase and increase the number of Mo_2_FeB_2_ phases as well as refining the grains of the Mo_2_FeB_2_ phase. In addition, according to the solidification theory, nucleation work Δ*G* and nucleation rate *N* can be obtained by Equations (1) and (2) [30].

(1) $$\Delta G=\frac{16}{3}\pi \left(\frac{\sigma_{LS}^{3}}{\Delta G_{m}^{2}}\right),$$

(2) $$N=N_{0}exp\left(-\frac{\Delta G}{kT}\right),$$

where, $\sigma_{LS}$ represents solid-liquid interfacial tension; Δ*G_m_* is the solid-liquid unit volume free energy difference; *k* is the boltzmann constant; *N*_0_ is constant and *T* is temperature of the liquid metal, respectively.

The addition of Ce, Y active elements reduce solid-liquid interfacial tension (σ*_LS_*). According to Equation (1), the nucleation work Δ*G* also decreases. Therefore, more and more liquid atoms reach the nucleation work through energy fluctuations, which improves the nucleation rate *N* according to Equation (2). In addition, during solidification of weld pool, rare-earth Y is enriched in the front of the solid-liquid interface due to the limitation of diffusion [31], the inter metallic compounds with high points containing rare-earth Y are dispersed at grain boundaries [31], which hinders the growth of the Mo_2_FeB_2_ nucleus and refines the Mo_2_FeB_2_ grain size. At the same time, Ce, Y active elements increase the fluidity of the liquid metal [32] and reduce the component supercooling during solidification as well as decrease the component segregation [33] to homogenize the structure.

**Figure 9:**
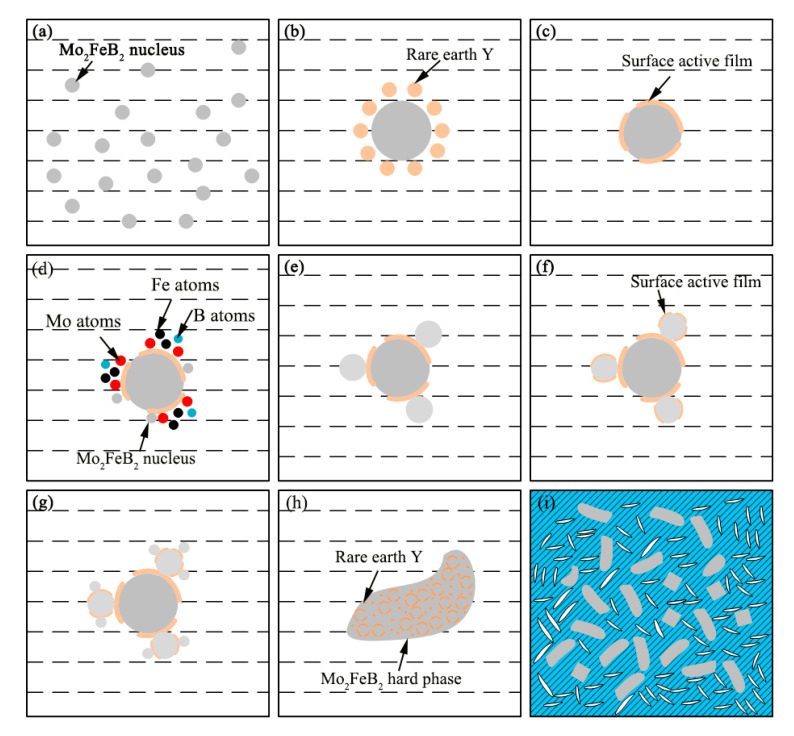
Schematic diagram of rare-earth action mechanism. (**a**) Nucleation process of Mo_2_FeB_2_; (**b**) Diffusion process of Y element; (**c**) Formation process of surface-active film; (**d**) Enrichment behavior of Mo, Fe and B atoms; (**e**) Reformation process of Mo_2_FeB_2_ particles; (**f**) Reformation process of surface-active film; (**g**) Reformation process of Mo_2_FeB_2_ particles; (**h**) Growth process of Mo_2_FeB_2_ hard phase; (**i**) Final microstructure.

Figure 9 shows the schematic diagram of rare-earth action mechanism. In Figure 9a, according to previous analysis, the Mo_2_FeB_2_ nucleus forms according to two types of forming methods (L→Mo_2_FeB_2_, 2MoB + Fe→Mo_2_FeB_2_). Meanwhile, rare-earth Y rapidly diffuses around the Mo_2_FeB_2_ particles, as shown in Figure 9b, where a hard-phase nucleus grows. Y, surface active element, acts as a surface-active film formed on the interface of solid and liquid and also hinders the diffusion of Mo, Fe and B atoms in the liquid metal to the Mo_2_FeB_2_ nucleus (Figure 9c). Thus, the growth of Mo_2_FeB_2_ grains is restricted. In addition, Y reduces solid-liquid interfacial tension (σ*_LS_*) and decreases the specific surface energy. Mo atoms, B atoms and Fe atoms are enriched in the surface of the nucleus with Y and the Mo_2_FeB_2_ nucleus precipitated from liquid is also enriched in the surface, as shown in Figure 9d. In Figure 9e, MoB react with Fe to form Mo_2_FeB_2_ particles when the composition, energy and structure requirements are met, the newly grown Mo_2_FeB_2_ surface is enriched in the active film containing Y again and the growth of Mo_2_FeB_2_ grains is restricted. Repeating this process, the Mo_2_FeB_2_ hard phase grows until the liquid metal cools to the eutectic temperature, as shown in Figure 9h. At last, all the remaining liquid phase is converted to Mo_2_FeB_2_, MoB, CrB and FeCr matrix, as shown in Figure 9i.

### 3.4. Wear Characteristics

**Figure 10:**
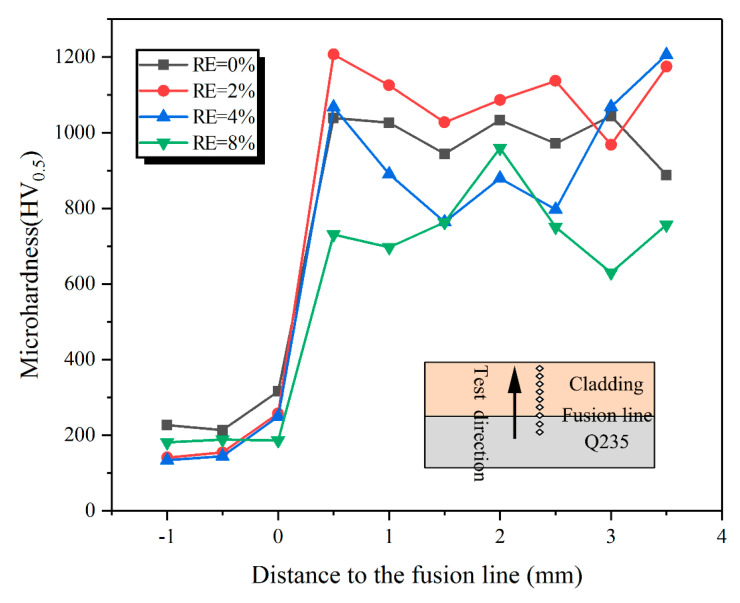
Microhardness of claddings with different RE contents.

Figure 10 shows average microhardness from substrate to the claddings. The average microhardness of the claddings with 0%, 2%, 4% and 8% RE are about 992 ± 59 HV, 1078 ± 105 HV, 929 ± 165 HV and 743 ± 100 HV, respectively, which is 4~6 times that of the substrate (180 HV), and the average microhardness of sintered samples is 925.5 HV.

**Figure 11:**
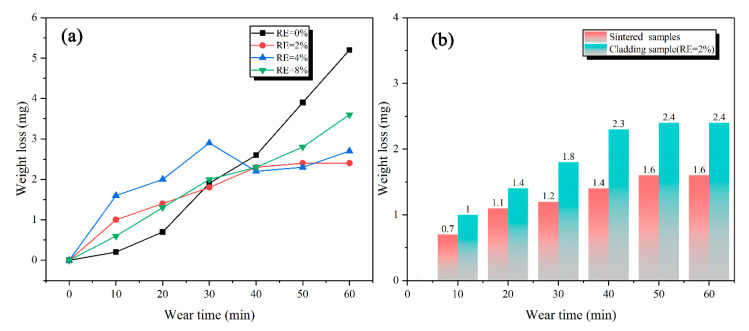
Wear weight-loss of claddings with different RE contents and sintered sample. (**a**) Wear weight-loss of claddings with different RE contents; (**b**) comparison of the wear weight-loss between the sintered sample and cladding with 2% RE content.

Figure 11 shows the relationship curves between the wear weight-loss and wear time with different RE content and sintered samples. The wear weight-loss of claddings with 0% and 2% RE content have a tendency to gradually increase and the former is significantly higher than the latter. This is because the addition of rare-earth elements forms the more ternary borides Mo_2_FeB_2_, which serves as a wear-resistant framework and effectively ensures the wear resistance of the deposited metal. At the same time, there is enough Fe-based solid solution in the cladding, which guarantees sufficient hardness in the material with certain toughness. The advantages of the two sides are fully combined to improve the wear resistance of the cladding. However, when the RE content is 0%, the amount of ternary boride Mo_2_FeB_2_ is insufficient (the area fraction of is 59%) and the grain size of Mo_2_FeB_2_ hard phase is relatively coarse (the grain size of is 16.52 μm). Thus, the increase of wear weight-loss is larger as wear progresses. When the RE content is 8%, although there is enough ternary boride as wear-resistant framework, an excessive amount of ternary boride Mo_2_FeB_2_ formed (the area fraction of is 74%) and part of the Mo_2_FeB_2_ hard phases adhered and segregated with each other as well as in a heterogeneous distribution (Figure 3d), which easily caused Mo_2_FeB_2_ hard phases to fall off during the wear process and aggravated the wear process in turn. Compared with the cladding with 0% and 8% RE content, sufficient ternary boride Mo_2_FeB_2_ (the area fraction of is 72%) is finely and uniformly distributed in the cladding (the average grain size is 4.78 μm) when the RE content is 2%, thereby showing excellent wear resistance (2.4 mg). Figure 11b shows the comparison of the wear weight-loss between the sintered sample and cladding with 2% RE content. The wear weight-loss of the sintered sample is 1.6 mg after 60 min while the counterpart of cladding with 2% RE content is 2.4 mg, which is about 70% of the wear resistance of the sintered sample.

**Figure 12:**
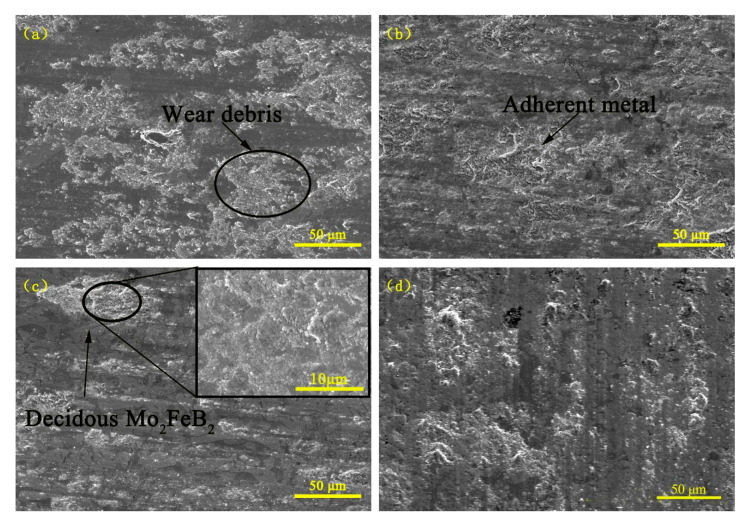
Wear morphology of claddings with different RE contents: (**a**) RE = 0%; (**b**) RE = 2%; (**c**) RE = 8%; (**d**) sintered sample.

Figure 12 shows the wear surface morphologies of the sintered sample and the claddings with 0%, 2% as well as 8% RE content for 60 min. From Figure 12a, there apparently exists wear debris on the wear surface, this is because the average grain size of the Mo_2_FeB_2_ phases is larger (16.52 μm) and the average free path of the matrix is also larger, which makes the matrix spacing between ternary borides increase and the abrasive particles are easily able to damage the matrix to form wear debris. When the RE content is 2% (Figure 12b), there is some adherent metal next to the hard phases. Research shows that adding proper rare-earth Y elements to the deposited metal can refine the structure and improve the toughness. Under the condition of multiple-impact wear, the plastic deformation occurs to the wear surface, which delays the crushing process and improves the wear resistance [34]. Therefore, the Mo_2_FeB_2_ phase is finely and uniformly distributed in the cladding when the RE content is 2%, the spacing between Mo_2_FeB_2_ becomes smaller, that is, the average free path of the matrix is smaller and the boride prevents the abrasive particles from damaging the matrix, which makes the wear resistance of cladding improved. At the same time, the boride and the matrix structure are mutually protected and Mo_2_FeB_2_ functions as wear-resistant framework. However, when the RE content reaches 8% (Figure 12c), there are too many Mo_2_FeB_2_ phases and some of them are connected to each other (Figure 3d), which reduces the fluidity of the liquid molten pool. Although the average free path of the matrix is small, the boride and the matrix structure cannot mutually protect due to the absence of the Fe-based solid solution. The bonding strength between hard phases and the matrix is reduced, Mo_2_FeB_2_ phases drop down during the wear process and the fallen hard phases become abrasive to aggravate the wear, which corresponds to results of the wear weight-loss (Figure 10). As for the sintering sample (Figure 12d), whose wear morphology is similar to the cladding with 2% RE content, only a small amount of plastic deformation appears on the wear surface.

## 4. Conclusions

1. Rare earth Y has a good refinement effect on Mo_2_FeB_2_ grains. When the RE content is 2%, average grain size of Mo_2_FeB_2_ phase is 4.78 μm, which is 1/3.44 of the cladding with 0% RE content.
2. Rare earth Y rapidly diffuses around Mo_2_FeB_2_ particles and serves as surface active film to hinder the growth of the Mo_2_FeB_2_ nucleus. Meanwhile, Mo atoms, B atoms and Fe atoms are easily enriched in the surface active film to form the Mo_2_FeB_2_ nucleus, which increases the number of Mo_2_FeB_2_ phases as well as refining Mo_2_FeB_2_ grains. In addition, rare earth Y reduces the specific surface energy and more liquid atoms reach the nucleation work through energy fluctuations, so that the nucleation rate of Mo_2_FeB_2_ is also improved.
3. The addition of rare-earth Y element plays an important role in improving the wear resistance of cladding. When the RE content was 2%, the wear resistance of cladding was 2.4 mg, which is about 70% of the sintering sample with similar wear-track morphology.

## Figures and Tables

**Table 1:** Technical parameters of the alloy powders.

Powder	Mean Particle Size (μm)	Chemical Composition (wt.%)
Mo	100	Fe < 0.002, O < 0.1, Si < 0.001, Bal Mo
Fe	90	C < 0.1, N < 0.1, O < 0.2, Bal Fe
Cr	110	O < 0.2, Fe < 0.18, N < 0.045, Bal Cr
FeB	60	B = 22%, C < 0.27, Si < 0.71, Bal Fe
RE	100	Mg = 8%, RE = 8.1% (Y = 4.2%), Si = 41%, Bal Fe

**Table 2:** Compositions of the alloy blocks (wt.%).

Samples	Mo	Cr	B	RE	Fe
No.1	47.5	10	6	0	Bal.
No.2	47.5	10	6	2	Bal.
No.3	47.5	10	6	4	Bal.
No.4	47.5	10	6	8	Bal.

**Table 3:** Technological parameter of carbon arc surfacing.

Carbon Rod Size/mm	Polarity	Swing Way	Voltage/V	Current/A	Welding Speed mm/min
Φ8 × 300	DC-positive connection	Rectangular swing	20~25	230~250	100

**Table 4:** Composition of hard-phase and eutectic structure of cladding with 2% RE content.

Position	Structure	Element Mass Percentage (wt.%)			
		Mo	Fe	Cr	B
Point 1	Hard phase	56.27	14.01	4.28	25.44
Point 2	Eutectic structure	10.77	49.54	9.07	30.61
